# Ibandronate Suppresses Changes in Apatite Orientation and Young's Modulus Caused by Estrogen Deficiency in Rat Vertebrae

**DOI:** 10.1007/s00223-021-00940-2

**Published:** 2022-01-06

**Authors:** Takuya Ishimoto, Mitsuru Saito, Ryosuke Ozasa, Yoshihiro Matsumoto, Takayoshi Nakano

**Affiliations:** 1 Division of Materials and Manufacturing Science, Graduate School of Engineering, Osaka University, 2-1 Yamadaoka, Suita, Osaka 565-0871 Japan; 2 Department of Orthopaedic Surgery, The Jikei University School of Medicine, 3-25-8 Nishi-Shinbashi, Minato-ku, Tokyo 105-8461 Japan; 3 Product Research Department, Kamakura Research Laboratories, Chugai Pharmaceutical Co., Ltd., 200 Kajiwara, Kamakura, Kanagawa 247-8530 Japan

**Keywords:** Ibandronate, Osteoporosis, Bone quality, Material anisotropy, Apatite orientation, Mechanical integrity

## Abstract

Bone material quality is important for evaluating the mechanical integrity of diseased and/or medically treated bones. However, compared to the knowledge accumulated regarding changes in bone mass, our understanding of the quality of bone material is lacking. In this study, we clarified the changes in bone material quality mainly characterized by the preferential orientation of the apatite *c*-axis associated with estrogen deficiency-induced osteoporosis, and their prevention using ibandronate (IBN), a nitrogen-containing bisphosphonate. IBN effectively prevented bone loss and degradation of whole bone strength in a dose-dependent manner. The estrogen-deficient condition abnormally increased the degree of apatite orientation along the craniocaudal axis in which principal stress is applied; IBN at higher doses played a role in maintaining the normal orientation of apatite but not at lower doses. The bone size-independent Young's modulus along the craniocaudal axis of the anterior cortical shell of the vertebra showed a significant and positive correlation with apatite orientation; therefore, the craniocaudal Young’s modulus abnormally increased under estrogen-deficient conditions, despite a significant decrease in volumetric bone mineral density. However, the abnormal increase in craniocaudal Young's modulus did not compensate for the degradation of whole bone mechanical properties due to the bone loss. In conclusion, it was clarified that changes in the material quality, which are hidden in bone mass evaluation, occur with estrogen deficiency-induced osteoporosis and IBN treatment. Here, IBN was shown to be a beneficial drug that suppresses abnormal changes in bone mechanical integrity caused by estrogen deficiency at both the whole bone and material levels.

## Introduction

Osteoporosis is associated with increased fracture risk owing to changes in bone quality and quantity. Bone quality is defined as the material, architectural, and mechanical characteristics, other than quantity, which contribute to bone strength. Despite extensive research on bone quantity, there is limited literature regarding bone quality in osteoporosis. Therefore, there is a need to investigate bone quality in osteoporosis and its treatments.

To prevent and treat osteoporotic disorders, various agents, such as bisphosphonates, parathyroid hormone, selective estrogen receptor modulators, receptor activator of NF-κB ligand inhibitor, and calcitonin, have been developed. Anti-osteoporotic agents have unique functions in controlling bone metabolism depending on their action mechanisms; therefore, there may be bone changes that cannot be detected from changes in bone mineral density (BMD), which is used as a gold standard in the diagnosis of osteoporosis. Given that combined administration of anti-osteoporotic agents has also become a promising option [1], it is important to understand the effects of each drug on bone characteristics other than BMD.

In the recently growing market for anti-osteoporotic agents, bisphosphonates have continuously held the largest share [2], and are used worldwide as effective therapeutic agents [3]. As the number of patients receiving bisphosphonate treatments continues to grow [4], it is essential to determine their impact on bone quality. Ibandronate (IBN) is a nitrogen-containing bisphosphonate that is beneficial for the treatment of postmenopausal osteoporosis, glucocorticoid-induced osteoporosis, Paget’s disease of bone, hypercalcemia of malignancy, and metastatic bone disease [5]. IBN is a highly potent inhibitor of bone resorption and is widely used clinically because it can be administered either orally or intravenously at extended between-dose intervals [6].

The main purpose of osteoporosis treatment is to maintain or increase bone strength, and thus reduce fracture risk. Bone strength is determined by a combination of bone mass, geometry, and material (tissue) properties [7], the latter two of which closely involve components of bone quality indices. The areal BMD measured by dual-energy X-ray absorptiometry (DXA) is influenced by bone mass, geometry, and volumetric BMD (vBMD); hence, DXA-BMD might conceal the changes in bone quality. In this study, we focused on bone material properties as indices of bone quality. Many bone quality indicators at the tissue level, such as microdamage accumulation [8–10], the degree of mineralization [8, 11], mineral crystallinity [12], and advanced glycation end-product cross-links [9, 13], have been proposed so far. As a promising bone material quality parameter, we propose and prove the importance of the crystallographic orientation of apatite crystallites and collagen molecular fibers in bone pathology [14, 15]. Unlike the other indices, the collagen/apatite orientation is unique owing to the fact that it is a kind of vector quantity and can describe the “anisotropic” features of bone material [16]. In intact bones, the crystallographic *c*-axis of apatite is aligned almost parallel to the direction of collagen fiber because of the epitaxial crystallization of apatite on the collagen template [17], resulting in the formation of an oriented nanocomposite. As a result, the stronger direction of apatite [18] corresponds to that of collagen [19], which makes the bone material property anisotropic along with a preferentially oriented direction. This is very important for bones that work in anisotropic stress fields. In particular, vertebral bodies are principally loaded parallel to the craniocaudal axis [20]. By using collagen/apatite orientation as an index, bone characterization along a specific direction (principal stress direction) is possible, and bone features that cannot be estimated from BMD, which is a scalar quantity, are expected to be clarified. Using the apatite orientation would be beneficial for the evaluation of osteoporosis pathology and medicinal effects of therapeutic agents for osteoporosis.

In the present study, we investigated the effects of IBN administration and its dose-dependence on the apatite *c*-axis orientation as bone material quality. Since the *c*-axis of apatite lies parallel to the collagen fiber direction as mentioned above, the degree of apatite *c*-axis orientation mirrors that of collagen fiber orientation [21, 22]; in other words, the apatite *c*-axis orientation indirectly—but essentially—indicates collagen orientation. The apatite crystalline size, which is included in the mineral crystallinity parameters [12], vBMD, and Young’s modulus were also analyzed. In addition, the mechanical properties at the whole bone level were measured.

## Materials and Methods

### Animal Experiments

Forty-two female Wistar–Imamichi rats were purchased from the Institute for Animal Reproduction (Ibaraki, Japan). Seven animals were sham-operated and the other thirty-five animals were ovariectomized (OVX) at eight months of age to develop a model for the study of estrogen deficiency. The experimental protocol was approved by the Institutional Animal Care and Use Committees of Ina Research Inc. and Chugai Pharmaceutical Co., Ltd. The animals were allowed free access to feed (CE-2; CLEA Japan, Tokyo, Japan) in stainless steel feeders, and drinking water was provided to the animals ad libitum via an automatic watering system.

**Fig. 1:**
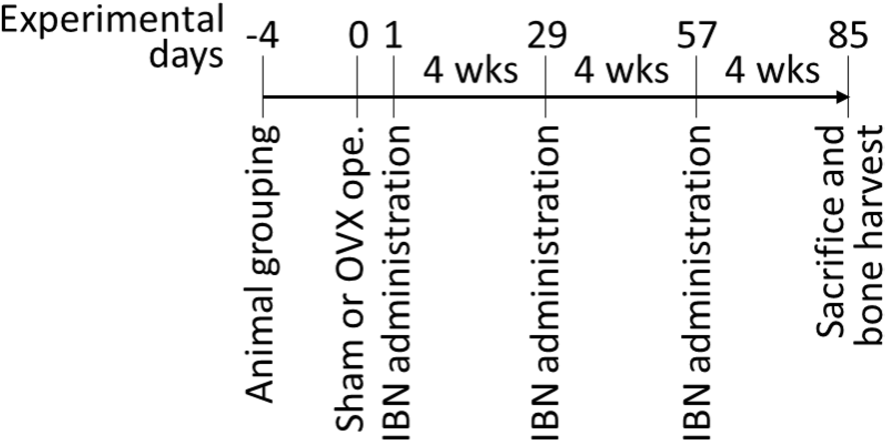
Protocol of animal experiment

From the day after surgery, twenty-eight OVX rats were treated with IBN (subcutaneously, once every four weeks, three times in total; 1, 3, 10, and 30 μg/kg (*n* = 7 in each dose group)) for 12 weeks (Fig. 1). Isotonic sodium chloride solution (Otsuka Pharmaceutical Factory, Tokushima, Japan) was administered to the other seven OVX rats to prepare the vehicle group. Body weights were measured on days − 4, 1, 29, 57, and 85. At 12 weeks after treatment (day 85), the lumbar vertebrae were collected and stored in 70% ethanol.

### Measurement of Trabecular Bone Structural Properties

Micro-computed tomography (μCT) (SMX-100CT; Shimadzu, Kyoto, Japan) was performed at 47 kV and 95 μA to produce bone images with a spatial resolution of 15 μm on each side to analyze the trabecular bone structure. The threshold for bone extraction was defined as a μCT gray value at which the gray value histogram showed a local minimum between the peaks representing bone tissue and soft tissues, including air. Trabecular bone from the central region of the fourth lumbar (L4) vertebra (1/4 of the total L4 height) was extracted, and the bone volume fraction (defined as bone volume/total volume (BV/TV)), trabecular thickness (Tb.Th), trabecular number (Tb.N), trabecular separation (Tb.Sp), the degree of anisotropy of trabecular bone architecture (DA), and structure model index (SMI) were analyzed [23] using TRI/3D-BON software (Ratoc System Engineering, Tokyo, Japan).

### Analyses of Apatite *c*-Axis Orientation and Apatite Crystallinity in Cortex

**Fig. 2:**
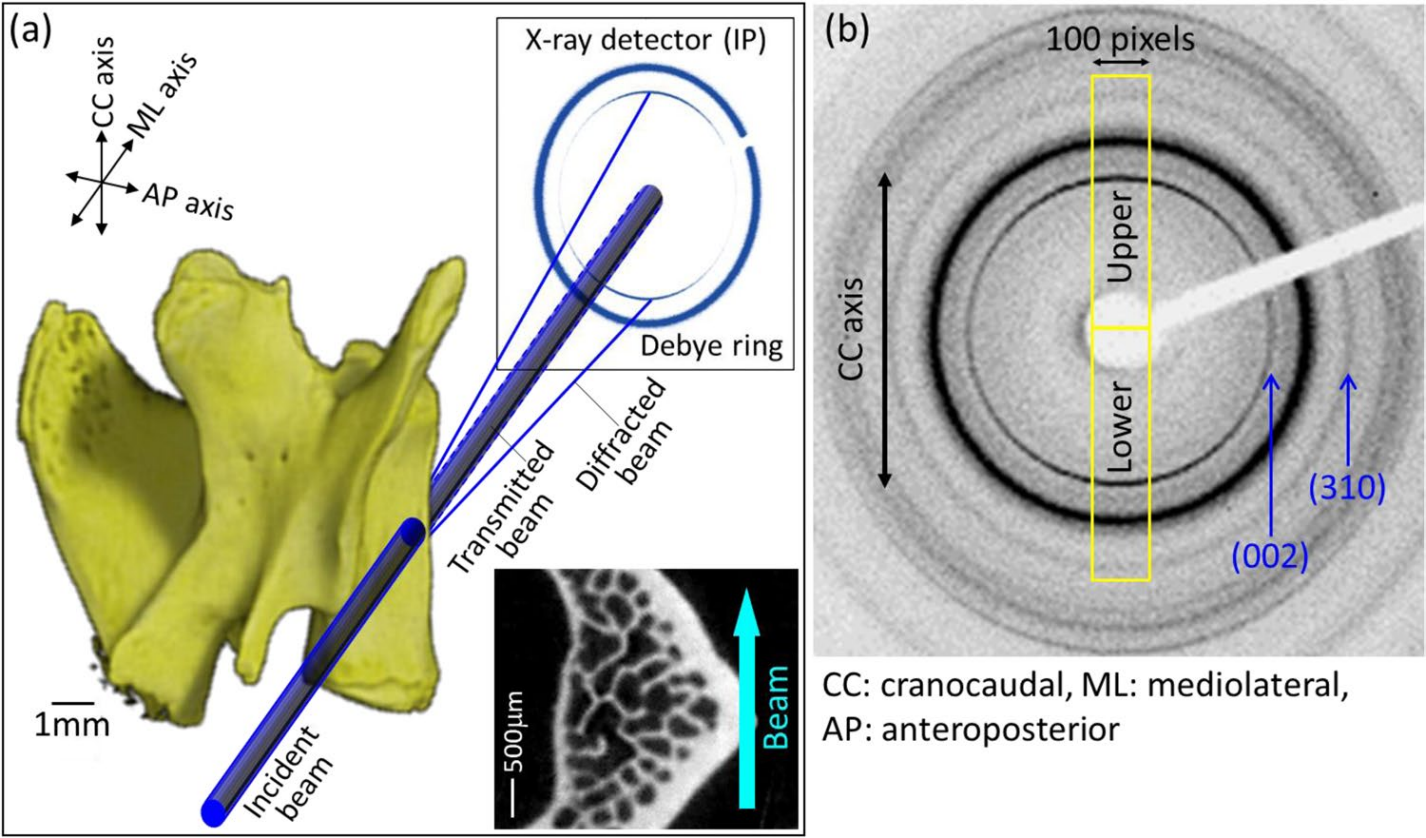
Microbeam X-ray diffractometer (μXRD) analysis of the degree of apatite *c*-axis orientation. **a** Schematic drawing of optical system with bone specimen and **b** a typical obtained μXRD pattern (Debye rings)

The degree of the apatite *c*-axis orientation in the L4 lumbar vertebral cortex was analyzed using a microbeam X-ray diffractometer (μXRD) system (R-Axis BQ; Rigaku, Tokyo, Japan) equipped with a transmission-type optical system and an imaging plate (storage phosphors) (Fuji Film, Tokyo, Japan) placed behind the specimen. Mo-Kα radiation with a wavelength of 0.07107 nm was generated at a tube voltage of 50 kV and tube current of 90 mA. The incident beam, collimated to a diameter of 200 μm, was radiated onto the middle anterior cortex vertically to the craniocaudal axis of the vertebra (Fig. 2a) to detect diffraction information along that axis. The beam diameter was determined to be sufficiently smaller than the anterior cortical thickness for all specimens assessed in the μCT image. The diffraction data were collected for 600 s to obtain adequate diffraction intensity.

From the obtained diffraction intensity pattern (Debye ring) (Fig. 2b), the two representative diffraction peaks for apatite, (002) and (310), were used for apatite *c*-axis orientation analysis, as previously described [21]. In the lumbar vertebral cortex, the apatite *c*-axis preferentially orients along the craniocaudal axis [21]. Therefore, in this study, diffracted information along the craniocaudal axis was analyzed. The upper and lower parts of the Debye ring correspond to the craniocaudal axis. The diffraction intensities were azimuthally integrated in the range of 100 pixels to obtain an X-ray diffraction profile. The degree of preferential orientation of the *c*-axis in the apatite crystals was determined as the relative intensity ratio of the (002) diffraction peak to the (310) peak in the X-ray profile. This was previously reported as a suitable index for evaluating the degree of apatite orientation [16, 24, 25]. The intensity ratios calculated from the upper and lower parts of the Debye ring were averaged. Randomly oriented hydroxyapatite (NIST #2910: calcium hydroxyapatite) powder had an intensity ratio of 0.8; therefore, detected values > 0.8 indicated the presence of anisotropic apatite *c*-axis orientation in the analyzed direction.

The apatite crystalline size along its *c*-axis was estimated from the full width at half maximum (FWHM) of the (002) diffraction peak using Scherrer’s equation:

1 $$D=\frac{K\lambda }{B\mathrm{cos}\theta }$$

where *λ* is the wavelength, *K* is a constant equal to 0.9 [26], *B* is the FWHM in radians, and *θ* is the diffraction angle. Parameter *B* was corrected by:

2 $${B}^{2}={B}_{M}^{2}-{B}_{S}^{2}$$

where *B*_M_ is the diffraction line width and *B*_S_ is the default width of the instrument.

### Measurement of Volumetric Bone Mineral Density (vBMD) in the Cortex

The volumetric BMD (vBMD) of the L4 vertebral cortex was measured using peripheral quantitative computed tomography (pQCT) (XCT Research SA + ; Stratec Medizintechnik GmbH, Birkenfeld, Germany). The central cross section of the L4 vertebra along the craniocaudal axis was scanned at a resolution of 70 × 70 × 260 μm. The data for each voxel were exported in ASCII format, and the average vBMD was calculated using Microsoft Excel software based on the vBMD values for the individual voxels. The vBMD was calculated within the area approximately 200 μm from the tip of the anterior cortex. Because the spatial resolution of pQCT is not always adequate for examining small bone specimens, such as those from mice, the partial volume effect, which can lead to an underestimation of vBMD [27], needs to be carefully taken into consideration. In this case, to minimize the partial volume effect, only interior voxels were selected and averaged. The cortical bone was judged to be above a threshold value of 690 mg/cm^3^. The bone area was determined by counting the voxels with a vBMD of ≥ 690 mg/cm^3^.

### Analysis of Young's Modulus as a Tissue Level Mechanical Property in the Cortex

The L4 vertebrae were carefully cut perpendicular to the craniocaudal axis to expose the middle cross section using a circular saw (Model 660; South Bay Technology Inc., San Clemente, CA, USA) with a diamond wheel (0.30 mm thick and 100 μm diameter). The specimen cross-sections were polished to obtain a mirror surface for the nanoindentation measurements. After grinding with silicon carbide paper of progressively finer grit up to #2000 under deionized water, the specimen surfaces were then polished with a microcloth (Buehler Ltd., Lake Bluff, IL, USA) with a 0.05-μm alumina suspension. After specimen drying, Young's modulus was measured along the craniocaudal direction in the L4 anterior cortex using a nanoindentation system (ENT-1100a; Elionix, Tokyo, Japan) with a Berkovich diamond indenter. The Young’s modulus of bone has been reported to increase because of drying. However, the relationship between the relative magnitudes was confirmed to be consistent [28]. Five indentations were created, and the results were averaged. Load-depth measurements were performed on the specimen surface in accordance with the established conditions [29]. Briefly, the loading/unloading rate and maximum load were 400 μN/s and 6000 μN, respectively. To minimize the effects of the viscoelastic deformation behavior of the bone, which could help to prevent overestimation of the mechanical properties, a constant maximum load was held for 180 s before unloading [29]. All measurements also included a second constant load held for 30 s at 600 μN to establish the thermal drift rate and correct the data. The Poisson ratio of bone was assumed to be 0.3, and the region between 95 and 50% of the maximum load was used to calculate the slope of the unloading curve.

In this study, vBMD, the degree of apatite *c*-axis orientation, apatite crystalline size, and Young’s modulus were analyzed within the same region.

### Assessment of Whole Bone Mechanical Properties

To assess the mechanical properties of the vertebral bone, a compression test was conducted on the L5 vertebra. The vertebral arch and disk were removed from each bone, and the vertebral bodies were trimmed to a length of 5 mm on the craniocaudal axis. The trimmed vertebral bodies were placed in a bone strength tester (TK-252C; Muromachi Kikai, Tokyo, Japan), and the compressive strength was measured at a displacement rate of 2.5 mm/min. The crosshead displacement was recorded. From the load–displacement curve, the ultimate load (N), stiffness (N/mm), and energy (mJ) were obtained.

### Statistical Analyses

Quantitative results are expressed as mean ± standard deviation. The statistically significant difference from the sham or vehicle group was determined using two-tailed unpaired Student’s *t* test, followed by an *F* test for homoscedasticity. The significance of IBN dose-dependent changes was tested using one-way analysis of variance (ANOVA). Post-hoc Tukey’s honest significant difference or Games–Howell comparisons were conducted according to the test for homoscedasticity. Single and multiple regression analyses were performed for contribution analysis. Statistical significance was set at *p* < 0.05. SPSS version 25.0 J (SPSS Japan Inc., Tokyo, Japan) for Microsoft Windows was used for statistical analyses.

## Results

### Body Weight, Bone Size, and Trabecular Architecture

**Fig. 3:**
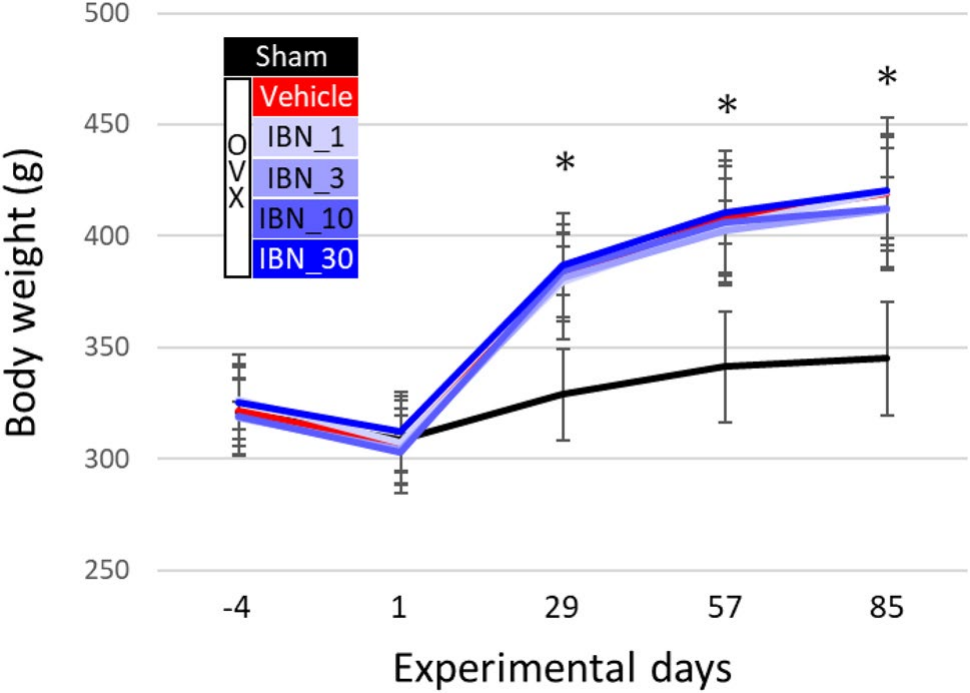
Change in body weight throughout experimental period (*n* = 7). *: *p* < 0.05 vs sham for all of OVX groups

**Fig. 4:**
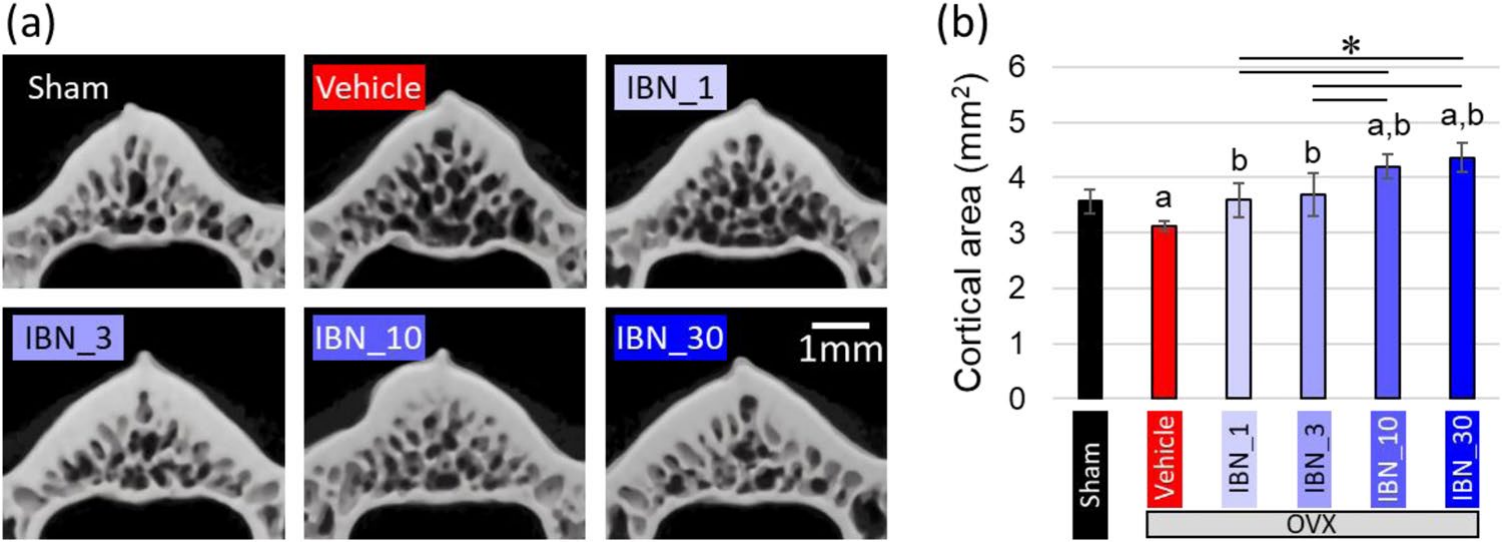
Change in bone mass of the fourth lumbar (L4) vertebral body. **a** Cross-sectional micro-computed tomography (μCT) images and **b** cross-sectional area of cortex at the center of the L4 vertebral body (*n* = 7). **a** *p* < 0.05 vs sham; **b** *p* < 0.05 vs vehicle; *p < 0.05

**Fig. 5:**
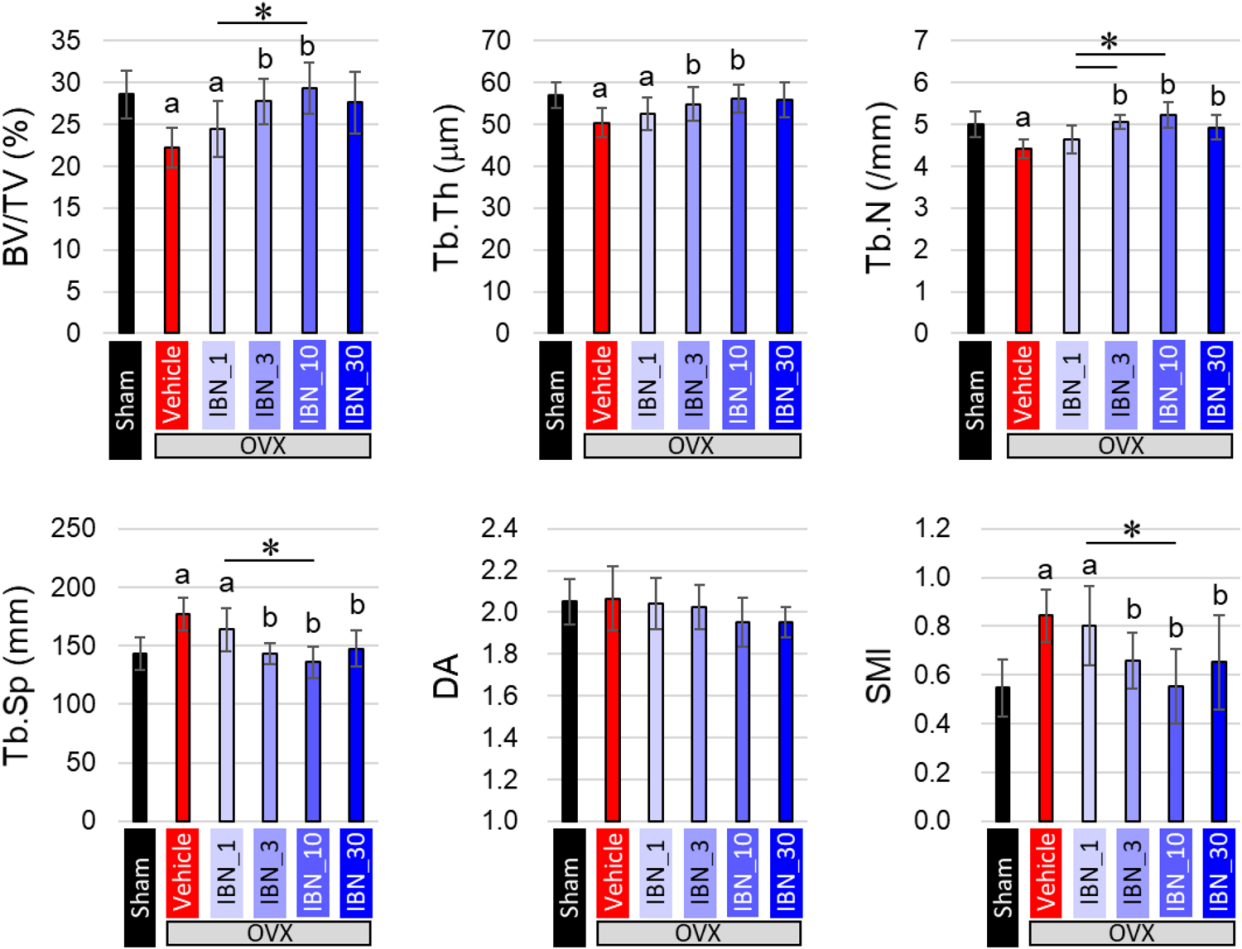
Architectural analysis of trabecular bone in the fourth lumbar (L4) vertebral body (*n* = 7). **a** *p* < 0.05 vs sham; **b** *p* < 0.05 vs vehicle; *p < 0.05

Estrogen deficiency significantly increased body weight, regardless of IBN administration (Fig. 3). This is due to an increase in adipose tissue [30]. Figure 4a shows the central μCT cross-section images of the L4 vertebral body. The cortical bone was thickened by the administration of IBN. The cortical cross-sectional area is shown in Fig. 4b. The outcomes of the architectural analysis of the trabecular bone are shown in Fig. 5. The vehicle group showed significant decreases in BV/TV, Tb.Th, and Tb.N and increases in Tb.Sp and SMI compared to those in the sham group, indicating trabecular bone loss and a morphological shift to a more rod-like shape. These changes are consistent with osteoporotic bone changes under estrogen-deficient conditions. Administration of IBN at 1 μg/kg (IBN_1) was insufficient for the suppression of bone loss and morphological change in trabeculae; however, administration of 3 μg/kg (IBN_3) and 10 μg/kg (IBN_10) significantly suppressed the osteoporotic changes induced by estrogen deficiency.

### Bone Material Characterization of Cortex

**Fig. 6:**
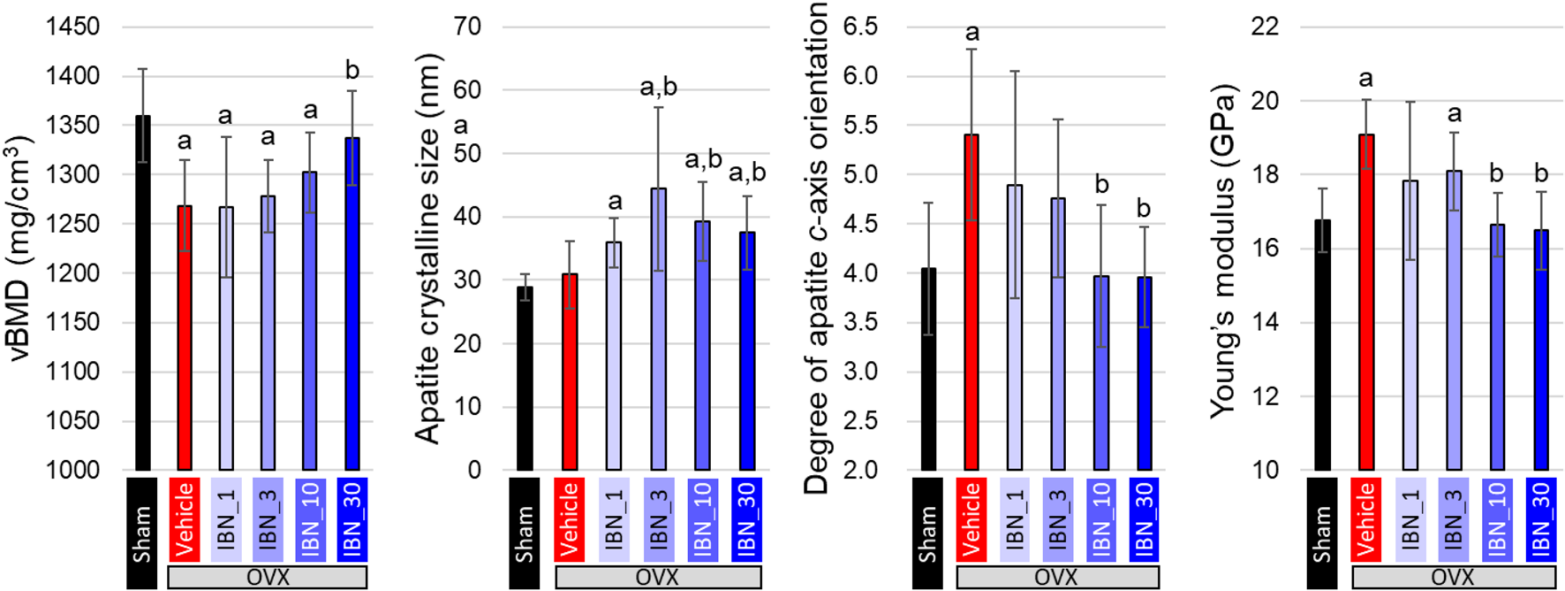
Variations in material properties analyzed in the fourth lumbar (L4) vertebral body cortex (*n* = 7). **a** *p* < 0.05 vs sham; **b** *p* < 0.05 vs vehicle

Figure 6 shows the variations in vBMD, apatite crystalline size along the *c-*axis*,* degree of apatite *c*-axis orientation, and Young’s modulus. The degree of apatite orientation and Young’s modulus were measured along the craniocaudal axis. The vBMD decreased in the vehicle group, whereas the administration of IBN at 30 μg/kg (IBN_30) prevented a decrease in vBMD. Estrogen deficiency did not change the crystalline size; in contrast, the administration of IBN significantly increased the crystalline size without dose dependency. The degree of apatite orientation along the craniocaudal axis was significantly increased in the vehicle group. The IBN_10 and IBN_30 groups showed the same degree of apatite orientation as the sham group. Interestingly, the apatite orientation changed in a manner opposite to that of vBMD. Young’s modulus, an important mechanical parameter at the bone material level, showed a similar tendency to the apatite orientation; the modulus was significantly increased in the vehicle group, and showed similar values in the IBN_10- and IBN_30-groups to that in the sham group.

**Fig. 7:**
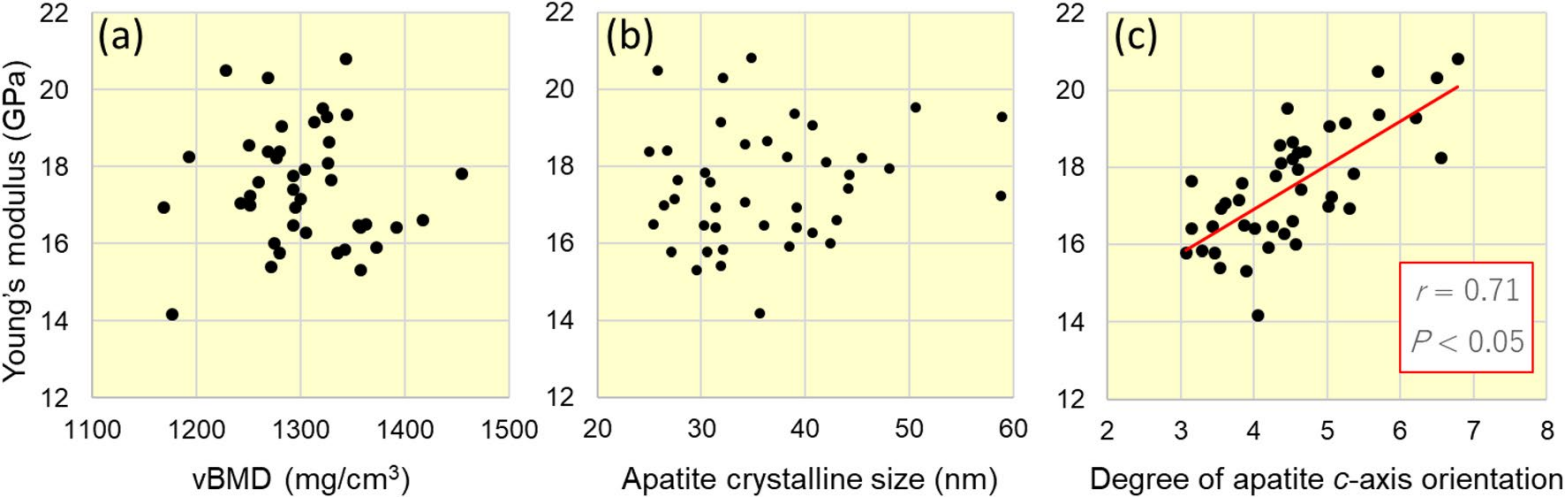
Correlation analyses between Young’s modulus and volumetric BMD (vBMD), apatite crystalline size, and degree of apatite *c*-axis orientation

From the single regression analyses, apatite orientation was significantly and positively correlated with Young’s modulus (*r* = 0.71, *p* < 0.05) (Fig. 7c), whereas vBMD and crystalline size were not (Fig. 7a, b). Multiple regression analysis (Table 1) derived multiple regression coefficients (*β*) and *p*-values of *β* = 0.08 and *p* = 0.50 for vBMD, *β* =  − 0.05 and *p* = 0.66 for apatite crystalline size, and *β* = 0.74 and *p* < 0.05 for apatite orientation, which further demonstrated the significant contribution of the apatite *c*-axis orientation to the bone material integrity.

**Table 1:** Relative contribution of volumetric BMD (vBMD), apatite crystalline size, and apatite orientation to Young’s modulus determined by multiple regression analysis

vBMD		Apatite crystalline size		Apatite orientation	
*β*	*P*	*β*	*P*	*β*	*P*
0.08	0.50	− 0.05	0.66	0.74	< 0.001

### Whole Bone Mechanical Properties

**Fig. 8:**
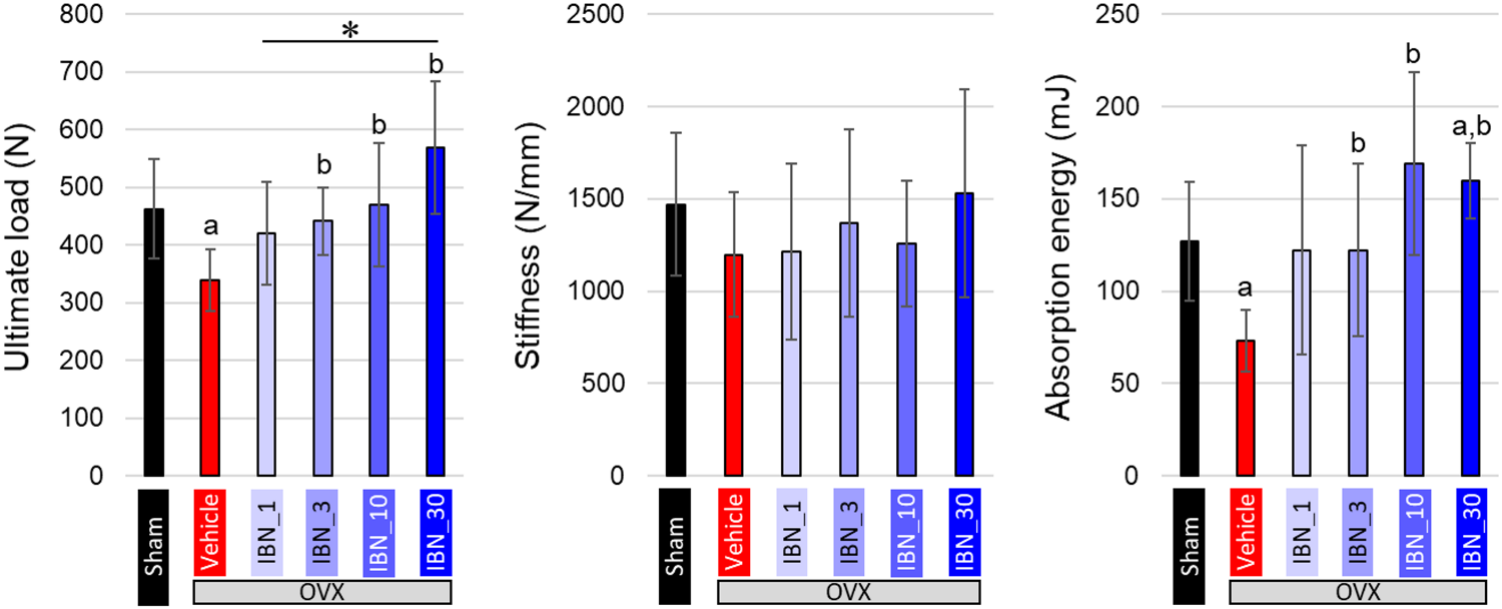
Variations in whole bone mechanical properties of fifth lumber (L5) vertebral body (*n* = 7). **a** *p* < 0.05 vs sham; **b** *p* < 0.05 vs vehicle; *p < 0.05

The stiffness, ultimate load, and absorption energy analyzed by the compression test are shown in Fig. 8. The ultimate load and absorption energy were decreased in the vehicle group; however, they increased to the same level as or higher than that in the sham group with the administration of IBN.

## Discussion

The dose-dependent effects of IBN for the prevention of estrogen deficiency-induced osteoporosis were examined in an OVX aged rat model for 12 weeks. Our aim was to investigate the bone quality and mechanical properties at the material level, in addition to the macroscopic bone architecture and whole bone strength. Significant changes in the preferential apatite orientation along the craniocaudal axis, which represents the anisotropy of bone material, can be reported here under estrogen-deficient and IBN-treated conditions. On the other hand, no significant difference was observed in the anisotropy of trabecular bone architecture.

IBN prevented a decrease in whole bone mechanical strength due to estrogen deficiency in a dose-dependent manner. The administration of 10 μg/kg IBN or more maintained a strength comparable to that in the sham group. This change in whole bone mechanical strength was synchronized with changes in bone area, BV/TV—which is equivalent to Tb.N × Tb.Th—and vBMD; therefore, it can be said that the prevention of bone loss by IBN played a role in maintaining the mechanical strength of the whole bone. These dose-dependent changes in strength and bone mass were consistent with previous reports [31, 32].

The material properties exhibited characteristic changes. vBMD showed a similar change with bone mass. However, the changes in apatite crystalline size and apatite *c*-axis orientation were completely different from those in bone mass. No change in apatite crystalline size was observed in the vehicle group with estrogen deficiency, whereas the administration of IBN significantly increased the apatite crystalline size. IBN binds to calcium ions and suppresses bone resorption by osteoclasts. The inhibition of bone resorption increases the maturity of apatite crystals [33]. In this study, crystal growth was demonstrated by the inhibition of bone resorption. The reported mineral/matrix ratio and increase in the degree of mineralization [33] might be partly due to crystalline growth. The degree of apatite orientation along the craniocaudal axis, in contrast, significantly increased under estrogen deficiency and decreased with IBN administration in a dose-dependent manner. Multiple regression analysis revealed that apatite orientation was the dominant factor controlling Young’s modulus. However, the increase in Young's modulus due to the increase in apatite orientation was not sufficient to suppress the decrease in whole bone mechanical strength under estrogen-deficient conditions because of the remarkable bone loss, resulting in reduced ultimate load and absorption energy in the vehicle group. This indicates that the maintenance of whole bone mechanical strength with IBN administration is largely due to the effective prevention of bone loss based on the suppression of bone resorption by osteoclasts.

However, discussing the material properties is important for fracture risk assessment. Abnormalities in the material properties reduce mechanical integrity regardless of bone mass [13]. The degree of apatite orientation shows a strong positive correlation (Pearson’s correlation coefficient *r* = 0.98) with that of collagen orientation in osteoporotic bone [21], and expresses the anisotropy of the bone extracellular matrix. The collagen/apatite orientation not only determines Young's modulus, as previously mentioned, but also strongly dominates the crack propagation behavior [34].

In this study, orientation and Young's modulus along the craniocaudal axis were increased in the absence of estrogen (vehicle group). However, it is necessary to discuss the mechanical properties in the directions other than the craniocaudal axis. Under estrogen deficiency-induced osteoporosis, the change in trabecular morphology (from plate to rod) [35] and the preferential disappearance of horizontal trabeculae [36], markedly reduced the mechanical strength in the horizontal direction (perpendicular to the craniocaudal axis). Homminga et al. [36] reported using μCT image-based finite element analysis that reduced horizontal trabeculae resulted in less resistance against uncommon loads. Their analysis did not consider material anisotropy (collagen/apatite orientation and Young’s modulus); therefore, it is conceivable that the osteoporotic vertebral body is actually less tolerant to uncommon loads than their calculations suggested. Furthermore, highly oriented collagen fibers effectively resist the propagation of cracks when they are oriented perpendicular to the crack propagation direction; conversely, when they are oriented parallel to the propagation, they facilitate it [34]. Collagen that is abnormally highly oriented in a certain direction does not have an effective mechanism that hinders crack propagation; thus, the crack propagates linearly and quickly. An excess degree of collagen/apatite orientation may be involved in fractures due to extraordinary and sudden loads, such as falls, in patients with osteoporosis.

There are three possible reasons for the increased apatite orientation in the vehicle group. The first is the direct effect of estrogen deficiency, the second is the effect of IBN, and the third is the increase in applied stress due to osteoporotic bone loss. All OVX-operated rats, including the IBN-treated rats, were deficient in estrogen, but the apatite orientation was less or not elevated in the IBN-treated groups, so the effect of estrogen deficiency was less evident. IBN suppresses bone resorption and increases crystallite size, but the increase in crystallite size does not necessarily affect the orientation, and the change in crystallite size does not actually correspond to the change in orientation, as shown in this study. Therefore, it is considered that the IBN hardly affects the orientation. However, regarding the applied stress, it is possible that the apatite orientation along the craniocaudal axis, which corresponds to the direction of principal stress, increased in response to the increase in body weight due to estrogen deficiency. The weight gain induced by estrogen deficiency was due to increased adipose tissue [30], and all of the OVX-operated groups gained a similar amount of weight. The cross-sectional area of the vertebral body increases depending on the dose of IBN, and the applied stress is determined by the balance between weight gain and change in cross-sectional area. To estimate the applied stress on the vertebra, the body weight/cross-sectional area was calculated [21], and its correlation with the apatite orientation was analyzed. A positive and moderate correlation with *r* = 0.55 and *p* < 0.05, was determined, suggesting that the magnitude of applied stress is one of the factors influencing apatite orientation. Thus, the increase in craniocaudal Young’s modulus (increased deformation resistance to stress) under estrogen deficiency would partly be the result of an adaptive response through which the bone managed to maintain strength even when osteoporotic bone loss was inevitable. Previous studies have shown a significant correlation between applied stress and apatite (collagen) orientation [21, 24], which is mediated by the mechanosensitivity of osteocytes [37]. In addition, reduced stress makes bone material less oriented (less anisotropic) [38, 39]. Taken together, stress applied to bone stimulates the formation of apatite orientation; however, further investigation is needed, using an in vivo quantitative loading model or finite element analysis, to determine the effect of applied stress on the bone material orientation under osteoporotic conditions.

To clarify the mechanical integrity of vertebral bone in estrogen-deficient and IBN-treated conditions, it would be important to analyze the mechanical properties and microstructure not only in the cephalocaudal axis but also in other direction than the cephalocaudal axis, because the strength in off-cephalocaudal axis reflects the resistance to uncommon loads caused by, for example, fall. In addition, since cortical and trabecular bone show different turnover rates and will be affected differently by estrogen deficiency and IBN treatment, it would be necessary to analyze the material quality, microstructure and mechanical properties of cortical and trabecular bone separately. These were not performed here, which is a limitation of this study.

It was shown that the material properties changed completely differently from the bone mass and whole bone strength. Our data revealed that IBN effectively suppressed bone abnormality due to estrogen deficiency in all of the indices measured, showing the benefit of IBN as an anti-osteoporosis agent. We believe that it is possible to elucidate the pathology of many types of bone disorders other than estrogen deficiency-induced osteoporosis, as well as the effects of therapeutic agents from a different perspective, using the collagen/apatite orientation index, which could lead to the development of better bone medication.

## Conclusion

In this study, we clarified the changes in bone material quality mainly characterized by the preferential orientation of the apatite *c*-axis associated with estrogen deficiency-induced osteoporosis and its prevention using IBN. The material properties changed completely differently from the bone mass and whole bone strength; however, IBN effectively prevented not only bone loss and the degradation of whole bone strength, but also abnormal increases in the degree of apatite orientation and Young’s modulus along the craniocaudal axis. Excess anisotropy in material properties leads to reduced mechanical integrity; therefore, IBN is beneficial for preventing the degradation of some material properties caused by estrogen deficiency-induced osteoporosis.
